# New Sustainable, Scalable and One-Step Synthesis of Iron Oxide Nanoparticles by Ion Exchange Process

**DOI:** 10.3390/nano11030798

**Published:** 2021-03-20

**Authors:** Ludovico Macera, Valeria Daniele, Claudia Mondelli, Marie Capron, Giuliana Taglieri

**Affiliations:** 1 Department of Industrial and Information Engineering and Economics, University of L’Aquila, Piazzale E. Pontieri 1, Monteluco di Roio, I-67100 L’Aquila, Italy; ludovico.macera@graduate.univaq.it (L.M.); giuliana.taglieri@univaq.it (G.T.); 2 CNR-IOM-OGG, Institut Laue Langevin, 71 Avenue des Martyrs, CEDEX 9, 38042 Grenoble, France; mondelli@ill.fr; 3 ESRF—The European Synchrotron, 71 Avenue des Martyrs, CEDEX 9, 38042 Grenoble, France; marie.capron@esrf.fr; 4 Partnership for Soft Condensed Matter (PSCM), ESRF—The European Synchrotron, 71 Avenue des Martyrs, CEDEX 9, 38042 Grenoble, France

**Keywords:** ion exchange process, δ-FeOOH nanoparticles, ferrihydrite nanoparticles, hematite nanoparticles, magnetite nanoparticles

## Abstract

This work introduces an innovative, sustainable, and scalable synthesis of iron oxides nanoparticles (NPs) in aqueous suspension. The method, based on ion exchange process, consists of a one-step procedure, time and energy saving, operating in water and at room temperature, by cheap and renewable reagents. The influence of both oxidation state of the initial reagent and reaction atmosphere is considered. Three kinds of iron nanostructured compounds are obtained (2-lines ferrihydrite; layered-structure iron oxyhydroxide δ-FeOOH; and cubic magnetite), in turn used as precursors to obtain hematite and maghemite NPs. All the produced NPs are characterized by a high purity, small particles dimensions (from 2 to 50 nm), and high specific surface area values up to 420 m^2^/g, with yields of production >90%. In particular, among the most common iron oxide NPs, we obtained cubic magnetite NPs at room temperature, characterized by particle dimensions of about 6 nm and a surface area of 170 m^2^/g. We also obtained hematite NPs at very low temperature conditions (that is 2 h at 200 °C), characterized by particles dimensions of about 5 nm with a surface area value of 200 m^2^/g. The obtained results underline the strength of the synthetic method to provide a new, sustainable, tunable, and scalable high-quality production.

## 1. Introduction

Among the transition metal oxides, iron oxides nanoparticles (NPs) are of technological and scientific importance because their relevant properties are size-dependent. In addition, their variety gives rise to a plethora of possible applications in different fields: from sensors and devices to medical diagnostic and treatments strategies, batteries, and catalysis, as well as in environmental remediation.

Specifically, iron compounds with oxygen are polymorphic in nature, having sixteen phases considering hydroxides, oxides, and oxyhydroxides [1]. The most relevant iron-based nanoparticles, from a technological point of view, are magnetite (Fe_3_O_4_), maghemite (γ-Fe_2_O_3_), hematite (α-Fe_2_O_3_), and ferrihydrite (Fe_5_HO_8_·4H_2_O), thanks to their unique properties, such as superparamagnetism, surface/volume ratio, great surface area, and easy separation methodology. The application areas of magnetite and maghemite NPs include medical applications (magnetic resonance imaging, antitumoral and hyperthermia), magnetic recording, and magnetic data storage devices; hematite NPs find application in lithium ion batteries, catalysis, coatings, gas sensors, transparent pigments, toners for xerography, and artificial photosynthesis; ferrihydrite NPs are successfully applied in wastewater treatments, sorbents, agricultural germination, and growth of maize [2,3,4,5,6,7,8,9].

Given the progress in these areas, it is fundamental to think in terms of large-scale production. At the same time, the consequences of a large-scale expansion of nanomanufacturing processes requires great attention, especially in terms of energy consumption, costs, time, and environmental impact. In order to take up the global challenge to realize large-scale productions compatible with the environment, much attention has to be focused on the preparation routes of the nanostructured products. To date, a lot of synthesis routes have been developed, starting both from chemical, physical, and biological methods. Specifically, the most used iron oxide NPs synthesis methods, due to their relative simplicity and the ability to have a good control of the particles’ shape and size, include chemical precipitation, hydrothermal, sol–gel, electro-deposition, emulsion precipitation, surfactant mediated precipitation, microemulsion precipitation, and microwave assisted hydrothermal technique [9,10,11,12,13,14]. Therefore, the main synthesis techniques are generally based on chemical routes, which, as disadvantages, often require the necessity of high temperature/pressure and always need extra purification steps to remove organic substances and/or secondary products. Such features lead to an unavoidable decrease of production yields, also increasing the times and costs to obtain the final pure product. For these reasons, to date, there is a lack of a method able to realize productions on large scale, which is of paramount importance for industrial applications, assuring a good control of the particles size and, at the same time, a desired not detrimental impact on the environment. In the present paper, we present an innovative, sustainable, and scalable one-step procedure to synthesize iron oxides NPs, allowing new perspectives for their availability on a large scale production (Patent application N.102019000017981).

Our main goal is to introduce a one-step synthesis method able to produce several kinds of iron-based NPs, with high yields, low costs, and time- and energy-savings, paying also a special attention towards a low environmental impact of the production process, thus limiting wastes and energetic costs of the process itself.

This innovative procedure is based on the ion exchange process, occurring in water, at room temperature and ambient pressure, between an anionic resin (OH^−^ form) and different iron chlorides. In appropriate conditions, the anionic resin absorbs chloride ions, rapidly releasing hydroxyl groups. The very rapid exchange between chlorides and hydroxyl groups was evaluated in our previous works [15,16,17,18], and the low solubility of iron hydroxide compounds brings to high supersaturation conditions, leading to a burst nucleation of the solid phase, whose growth is strongly limited due to the rapid consumption of hydroxide reservoir. This phenomenon can allow synthesizing pure iron oxides/oxyhydroxides NPs, dispersed in aqueous medium, properly tuned according to the selected initial iron reagent and reaction atmosphere. The pure NPs are obtained in a single step, without the need of any purifications or washing phases (as it is the case for the very popular coprecipitation techniques occurring at ambient temperature), with the advantage of reducing times and costs and strongly increasing the final production yields from grams-level to hundreds of pounds of annual production at lab-scale. Finally, it is important to also stress the fact that, at the end of the exchange process, the resin, removed by a sieving procedure, can be regenerated in order to be ready for a new production again. This last point is particularly important, making the process very low waste, and leading to an overall sustainable process, thanks to the low energy consumption and to the low overall environmental impact.

As reported in literature [19,20], although the ion exchange route was already employed to synthesize other oxides (i.e., tungsten trioxides and porous iron oxides), the novelty of our work consists in a very fast anionic exchange process, allowing obtaining pure iron oxide NPs without cations and anions impurities.

We studied the synthesized NPs, in terms of structure, crystallinity, morphology, size dimensions, and specific surface area by using several techniques, such as X-ray diffraction (XRD), transmission and scanning electron microscopy (TEM, SEM), atomic force microscopy (AFM), and surface area measurements with the Brunauer–Emmett–Teller method (BET).

## 2. Materials and Methods

### 2.1. Materials

Iron(III) chloride hexahydrate (FeCl_3_·6H_2_O) and iron(II) chloride tetrahydrate (FeCl_2_·4H_2_O) are supplied by Zeus (purity > 99%); ion-exchange resin Dowex Monosphere 550A is supplied by Sigma Aldrich (St. Louis, MO, USA), in the form of translucent spheres characterized by a particle size of 590 ± 50 µm, characterized by hydroxyl groups on its substrate (R-OH) and having a total volume capacity of 1.1 eq/L. Deionized water was purified by a Millipore Organex system (R ≥ 18 MΩ·cm, Merck KGaA, Darmstadt).

### 2.2. Synthesis of Iron Oxide or Hydroxide Nanoparticles by Ion Exchange Method

**Figure 1:**
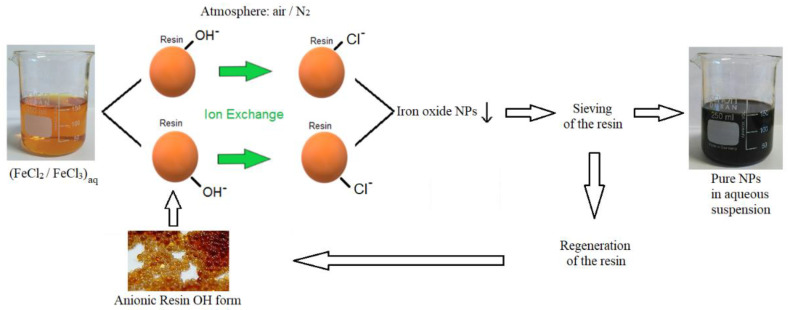
Graphical scheme of the ion exchange process.

Our original method of production starts from an aqueous solution of iron chlorides then mixed with a suitable amount of anionic resin, having hydroxyl groups on its substrate (R-OH). Specifically, three different solutions of initial reagents are prepared in relation to the iron oxidation state: (1) Fe(II), (2) Fe(III), and (3) a mix of Fe(III) and Fe(II) in a weight ratio of 2:1. For each preparation, the mixing procedure with the anionic resin is carried out under moderate stirring, at room temperature (20 °C) and ambient pressure, for only 10 min. At the beginning of the mixing, a solid phase rapidly appears. In order to determine if the environment of reaction affects the quality of the final product, each synthesis is performed both in air and in nitrogen as an inert atmosphere. For all the syntheses, in order to estimate the reagents consumption, we measured the reduction between the initial chloride concentration (CC) and the residual chloride concentration (RCC), after 1 and 10 min, (ΔCC). The chloride concentrations are specifically measured by means of an ion-sensitive electrode (Metrohm, Herisau, Switzerland). At the end of the synthesis, the aqueous NPs suspension is separated from the resin using an easy sieving procedure (mesh < 200 µm). In turn, the exhausted resin is regenerated by means of an 8% NaOH aqueous solution, and it will be reused for the successive production, according to a cyclic procedure. The graphical scheme of the synthesis process is shown in Figure 1.

In order to investigate the feasibility of this method to obtain not only a few grams of NPs, as obtained in our recent paper [18], we here considered operating with 0.5 moles of each initial iron chloride reagent.

Depending on the oxidation state of the initial reagent, we can expect different hydroxides from each synthesis, according to the following reactions [21]:

Fe(II): FeCl_2_ + 2R-OH → Fe(OH)_2_ + 2R-Cl (1)

Fe(III): FeCl_3_ + 3R-OH → Fe(OH)_3_ + 3R-Cl (2)

Fe(III) + Fe(II): 2FeCl_3_ + FeCl_2_ + 8R-OH → 2Fe(OH)_3_ + Fe(OH)_2_ + 8R-Cl (3)

However, in Reaction (1), we expected that the ferrous hydroxide, Fe(OH)_2_, develops only in an environment without oxygen, because in aerobic conditions the iron(II) will be rapidly oxidized by the protons of water to form iron(II, III) oxide and molecular hydrogen. This process is described by the Schikorr reaction [22]:

3Fe(OH)_2_ → Fe_3_O_4_ + H_2_ + 2H_2_O (4)

Additionally, under inert atmosphere, Fe(OH)_2_ could be not stable in presence of hydroxyl ions. Indeed, we can expect the following reaction [23]:

Fe(OH)_2_ + OH^−^ → FeOOH + H_2_O + e^−^ (5)

which leads to the formation of a polymorph of oxy-hydroxides compounds [24].

Concerning Reaction (2), the ferric hydroxide, Fe(OH)_3_, is unstable, because the molecules, formed during the hydrolysis of ferric solutions, interact to produce an amorphous ferric oxide hydrate precipitate, named ferrihydrite [14], according to the following reaction:

5Fe(OH)_3_ → Fe_5_HO_8_ 4H_2_O + 3H_2_O (6)

This iron oxy-hydroxide ferrihydrite is considered the first stable product of the hydrolysis of iron(III) ions in water. The ferrihydrite has different formulas depending on its crystallinity, from two-line ferrihydrite with very low crystallinity to the “well crystallized” six line ferrihydrite [1].

Finally, when both oxidation states of iron are present, the two iron hydroxides tend to combine to directly form magnetite, both in aerobic and anaerobic conditions:

2Fe(OH)_3_ + Fe(OH)_2_ → Fe_3_O_4_ + 4H_2_O (7)

In summary, by varying the oxidation state of the initial reagent and the atmosphere (air or N_2_), the syntheses lead to six different suspensions, from here named S1_N2_, S1_air_; S2_N2_, S2_air_; and S3_N2_, S3_air_, respectively. Specifically, the numbers 1, 2, and 3 are related to Fe(II), Fe(III), and mixed Fe(II) + Fe(III), and the subscripts N_2_ and air are related to the N_2_ inert atmosphere and air, respectively. However, from the previous discussion, three different kinds of iron compounds are expected, an iron oxyhydroxide (FeOOH), magnetite (Fe_3_O_4_), and ferrihydrite (Fe_5_HO_8_·4H_2_O), as summarized in Table 1.

By means of the expected chemical reactions, reported above, it is possible to evaluate the stoichiometric values of the moles that are produced for each compound, named DRY_stoich_. By measuring from each suspension, the real dry product obtained after the resin separation, named DRY_meas_, we can estimate the yields of production for each synthesis, Y, as follows:

(8) $$Y=\frac{{\mathrm{DRY}}_{\mathrm{meas}}}{{\mathrm{DRY}}_{\mathrm{stoich}}}\;\times \;100$$

### 2.3. Production of Hematite and Maghemite NPs by Means of Calcination

As already known in literature [18,25,26], most iron hydroxides and oxides can be oxidized to form other iron oxides as well. There are different ways to oxidize iron compounds; the most typical approaches consist in the reaction with oxidizing agents or in increasing the temperature, maintaining the compounds in air.

For these reasons, the iron compounds obtained from the ion exchange process can be considered as precursors to obtain other iron oxides nanoparticles. The calcination treatments are carried out for 2 h with a temperature ramp rate of 10 °C/min at different temperatures, up to 600 °C, to find the minimum temperature necessary to observe phase transformations, limiting the diffusive growth phenomena of the new formed nanoparticles.

From the six initial precursors reported in Table 1, we obtained different calcined samples, depending on the oxidation or on the dehydration phenomena occurring during the calcination treatment [27]. The calcined samples showing a complete phase transformation are reported, named the precursor samples, adding a subscript relating to the minimum temperature T to which they are obtained. In particular, the obtained samples are indicated as follows: S1_N2_200_, S1_N2_600_, S1_air_200_, S1_air_600_, S2_N2_200_, S2_N2_500_, S2_air_200_, S2_air_500_, S3_N2_200_, S3_N2_600_, S3_air_200_, and S3_air_600_, respectively.

### 2.4. Characterization Analyses

Phase identification, structure, and crystallinity of the obtained NPs are investigated by means of X-ray diffraction (XRD). XRD scans are recorded by a PANalytical X’Pert PRO apparatus (Almelo, Netherlands) with Cu-Kα radiation, equipped with a diffracted-beam monochromator and a PiXCel 2D detector. XRD patterns are recorded in the range 3–70 °2θ, with step size of 0.026 °2θ and time for step 10 s. X-ray data are fitted using the pseudo-Voight profile function and refined by means of Rietveld refinements [28]. The functional groups are investigated by attenuated total reflectance–Fourier transform infrared spectroscopy measurements (ATR–FTIR), by means of a spectrophotometer Thermo Nicolet Nexus (Thermo Fisher Scientific, Waltham, MA, USA); data are collected in the range 400–4000 cm^−1^. Scanning electron microscope (Gemini SEM 500, ZEISS, Oberkochen, Germany) and transmission electron microscope (Philips TEM CM100, Amsterdam, the Netherlands) as well as atomic force microscopy (AFM, Cypher Asylum Research available at the AFM platform of the PSCM, in tapping mode) are used to investigate dimensions and morphology of the obtained NPs or of their aggregates. Regarding TEM images, the particles’ size distribution is evaluated by using ImageJ software.

The samples are prepared in accordance with the standard procedures, working, if necessary, under a nitrogen atmosphere in order to avoid any reaction with the atmospheric O_2_.

As concerns the samples obtained from calcination, they are prepared directly, depositing the dry powders onto adapted sample holders.

The nitrogen adsorption measurements are carried out at 77 K, using a Micromeritics ASAP2000 system (Micromeritics, Norcross, GA, USA), utilizing Brunauer–Emmett–Teller (BET) method for the surface area estimation. For each sample, about 0.2 g of dry powder are outgassed for about 15 h at 150 °C (5 × 10^−3^ Torr) before performing the measure. The pore-size distribution is determined as well, considering the desorption branch of the isotherms with the BJH (Barett–Joyner–Halenda) method.

## 3. Results and Discussion

### 3.1. Iron Oxides NPs Obtained after the Exchange Process

We report in Table 2 the kinetics results, in relation to residual chloride content (RCC) as well as the ΔCC reduction between the initial chloride concentration and the chloride concentration after 1 and 10 min of the exchange process. The obtained values denote a reagents consumption > 96% in the first minute of the exchange process, which saturates at values higher than 99% after only 10 min, denoting a very fast reaction and residual chloride contents (RCC) values of about 20–30 mg/L.

From these first results, it is clear that, for all the syntheses, the substitution of –OH groups with chlorides ions (Cl^−^) on the resin substrate is extremely fast, and the process completes within 10 min, with a reduction in the chloride content always higher than 99%. Tangible and extraordinary high yield of production (Y), related to the expected compounds, is measured as well, with values from 92% up to 96%, as reported in the last column of Table 2, corresponding to an amount of product of about 40 g of NPs in 10 min for each preparation.

**Figure 2:**
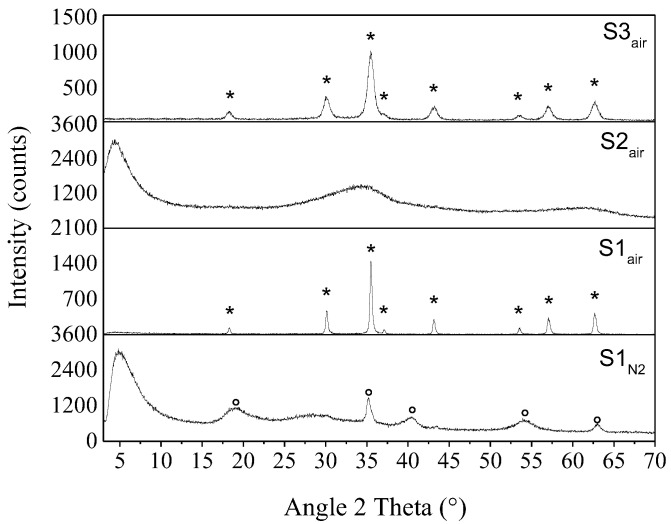
XRD patterns of dry powders of the samples obtained by the one-step synthesis. Legend: ° = δ-FeOOH (delta iron oxyhydroxide), * = Fe_3_O_4_ (magnetite).

XRD analyses allowed investigating the solid phase that formed after the exchange process. In particular, Figure 2 shows the XRD spectra of the obtained suspensions, revealing three different pure phases depending on the initial reagent or atmosphere, thus confirming the expected compounds reported in Table 1. Moreover, since from samples S2 and S3 we obtain the same results both in air and in N_2_, only the samples obtained in air are reported. In addition, the comparison between the XRD results, in terms of peaks list, and the ICDD reference patterns is reported in Figure S1.

However, specific observations must be underlined. The synthesis carried out starting from iron(II) ions in inert atmosphere, S1_N2_ sample, leads to the formation of δ-FeOOH (delta iron oxyhydroxide, ICDD pattern # 00-013-0518), characterized by a hexagonal crystal structure and showing a differential line broadening in the (001) Bragg peak, indicative of a platelet shape of diffraction domains [29,30]. The maximum at about 20 °2θ is very broad, probably related to some very small and poorly crystallized iron oxyhydroxide, consistent with the fact that δ-FeOOH never occurs as a single phase. By means of profile analysis, an average crystal grain size of about 6 nm is evaluated, as reported in Table S1. Moreover, a strong broad reflection at around 5 °2θ is observed, corresponding to a d-spacing of about 2 nm, which can be connected to an ordered mesoporous structure of this phase and to the presence of a uniform nanoparticle size, as previously discussed in literature [18,31,32]. As reported in [32], small particles could act as scattering centers, giving rise to scattering signal at low angles (SAXS peak) due to the particle size (10–100 Å). In fact, when the particles are very small and uniform in size, this SAXS peak is significantly more intense than any diffraction from the lattice planes.

In particular, as reported in Figure S2, during the synthetic route, performed in N_2_ atmosphere, the following transformation occurred: instantaneously after the addition of the iron(II) solution with the anionic resin, a green suspension is obtained (characterized by a pH value of 9.1). Then, after the lyophilization process (or other drying procedures), the obtained powder is red δ-FeOOH.

Contrarily, the synthesis performed starting from iron(II) ions but carried out in air, S1_air_ sample, reveals the formation of a crystalline phase attributed to well-defined Bragg peaks of cubic magnetite (Fe_3_O_4_, ICDD # 00-001-1111). Regarding the samples with only iron(III) ions, carried out both in inert atmosphere and in air (S2_N2_ and S2_air_ samples), they present the same diffraction pattern, characterized by the presence of two broad halos, peaked around 34 and 61 °2θ, respectively, attributable to the formation of the two-line ferrihydrite [14]. As in the S1_N2_ sample, we can observe the strong reflection at around 5 °2θ, but it results slightly shifted at lower 2θ angles, denoting a slightly larger distance between the particles into the mesoporous structure. Finally, from both the two syntheses starting from iron(II) and iron(III) ions, S3_N2_ and S3_air_ samples, a crystalline phase attributable to cubic magnetite, Fe_3_O_4_, ICDD # 00-001-1111, is shown. However, it is important to remark that the magnetite phase of S3_air_ sample is clearly characterized by broader Bragg peaks than the magnetite of S1_air_ sample, corresponding to an average crystallite size <D> of about 29 nm for S1_air_ sample and 8 nm for S1_air_ sample, respectively. This result could be ascribed to a more sudden and denser nucleation step in the samples characterized by the mixed Fe(II)/Fe(III) stoichiometry, leading to smaller crystal dimensions. For each sample, the crystallographic parameters of the synthesized phases as well as their average D values, evaluated by the Rietveld refinements, are reported in Table S1.

All these results allow confirming the formation of the compounds reported in Table 1. However, the XRD analyses underline the different crystal dimensions of the magnetite nanoparticles originating by different oxidation states and individuate the nanometric morphological features and the crystal structure of the specific oxyhydroxide compound. In particular, from the analysis of cell parameters, it is evident that the obtained cubic magnetite results are substoichiometric in relation to the lower value of their cell parameter with respect to the reference one (ICDD # 00-001-1111).

**Figure 3:**
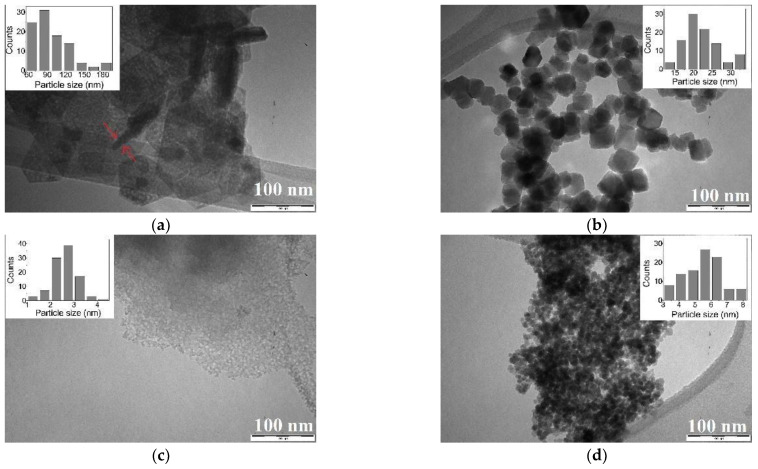
TEM images of the synthesize nanoparticles obtained from the different syntheses: (**a**) iron oxyhydroxide δ-FeOOH nanoparticles in S1_N2_ sample, with arrows underlining the thickness of lamellae; (**b**) magnetite nanoparticles in S1_air_ sample; (**c**) ferrihydrite nanoparticles of S2_air_ sample; (**d**) magnetite nanoparticles in S3_air_ sample.

In order to investigate size and morphology of the produced particles, we analyzed the samples by TEM (Figure 3). Confirming XRD results, the atmosphere of reaction (N_2_ or air) strongly affects Fe(II) samples, but it does not affect the phases in Fe(III) and Fe(II) + Fe(III) ones. For this reason, in the following figures, we report only the best representative images for S2_air_ and S3_air_ samples, showing that the particles of all the synthesized phases are in the nano-range and underlining the different dimensions and morphologies. Specifically, S1_N2_ sample, shown in Figure 3a, reveals a superimposition of extremely thin hexagonal lamellas, attributing to the iron oxyhydroxide δ-FeOOH phase, similarly to literature results but presenting a definite mesoporous structure as well [24,30]. In addition, from TEM observations, we found that the lamellas show a thickness of about 10 nm, as marked by the arrows. As concerns S1_air_ sample, it presents the typical cubic morphology of magnetite, with size dimensions of about 20 nm, as observed in Figure 3b, while S2_air_ sample, Figure 3c, appears constituted by a mesoporous aggregation of very small spherical particles of size dimension of about 2–3 nm, as typical of the 2-line ferrihydrite structure [33]. Finally, particles of about 6 nm constitute S3_air_ sample, Figure 3d, still representing the cubic morphology of magnetite but definitely smaller than that observed in S1_air_ sample, confirming the XRD results. In addition, if we compare the particle dimension, observed by TEM, with the crystallite size, evaluated by XRD measurements, we can assert that each nanoparticle in S1_air_ as well as in S3_air_ samples corresponds to a single crystal.

From Figure 3, we can also observe that, except for S1_N2_ sample where the hexagonal lamellas could derive from a self-assembling of primary nanoparticles [15,34,35], the other samples exhibit that the nanoparticles are monodisperse, as shown in the insets. This result can be strongly related to the fact that, during the synthesis, for all the samples a fast and diffuse nucleation occurs, and all the crystallites form at the same time and under the same environment, in the whole volume of reaction. Moreover, due to a very quick kinetic of the ion exchange process and due to a small solubility of the formed phases, a rapid substitution of the OH– sites on the resin occurs, limiting and controlling the growth rate of such crystallites as well.

**Figure 4:**
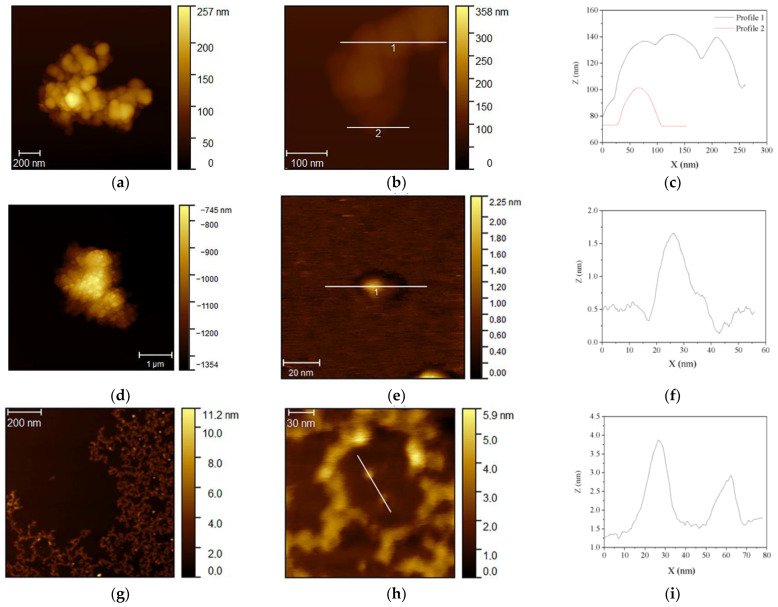
AFM observations of the nanoparticles obtained from the different one-step syntheses and corresponding profile analyses along *Z*-axis of single particles: (**a**–**c**) magnetite nanoparticles in S1_air_ sample; (**d**–**f**) ferrihydrite nanoparticles of S2_air_ sample; (**g**–**i**) magnetite nanoparticles in S3_air_ sample.

AFM observations of S1_air_, S2_air_, and S3_air_ samples are reported in Figure 4. For each sample, both representative images, at lower and higher magnification, and the corresponding profile analysis along the Z axis of single nanoparticles are shown. The analyses allow confirming the differences between the two synthesized magnetite nanoparticles coming from S1 and S3 samples, showing in both samples monodisperse particles, Figure 4a,g, but with height of about 20 nm in S1_air_ sample and 3 nm in S3_air_ sample, Figure 4b,c,h,i, respectively. As concerns the ferrihydrite S2_air_ sample, very close aggregates are observed (Figure 4d), probably due to the extremely fine monodisperse nanoparticles, with dimensions of about 2 nm (Figure 4e,f).

**Figure 5:**
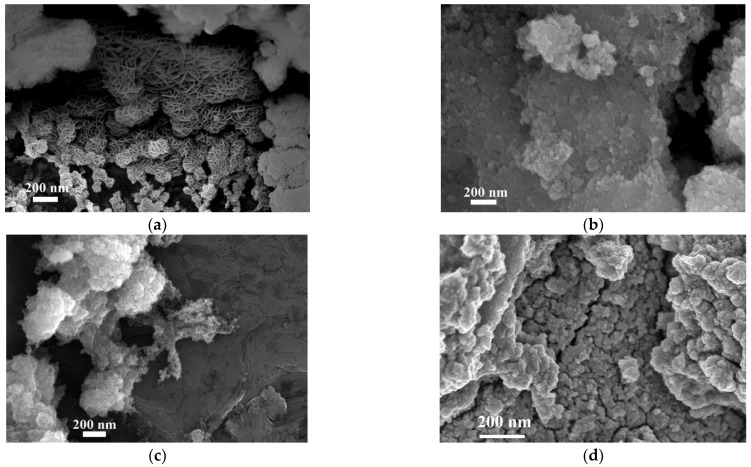
SEM images of the synthetized NPs, observed at a magnification of 100,000X, from different samples: (**a**) iron oxyhydroxide δ-FeOOH particles of S1_N2_ sample; (**b**) magnetite particles of S1_air_ sample; (**c**) ferrihydrite particles of S2_air_ sample; (**d**) magnetite particles of S3_air_ sample.

SEM images, shown in Figure 5, put in evidence the aggregation behavior, in relation to the different morphologies characterizing each sample. The δ-FeOOH sample S1_N2_ denotes spherical aggregations organized in an ordered network of nano-platelets, all having thicknesses < 20 nm, forming flower-like particles [35], as shown in Figure 5a. In addition, this network presents both macropores and mesopores, and it results in being characterized by a high whole porosity. The magnetite phases composing S1_air_ and S3_air_ samples are shown in Figure 5b,d, respectively. Considering the SEM resolution, they seem very similar, composed by a dense aggregation of small particles, and presenting a low porosity. However, the S3_air_ sample exhibits a denser nanoparticles aggregation probably due to the smallest dimensions observed by TEM and confirmed by XRD. A spongy, mesoporous, and spherically shaped assembly of very small nanoparticles confirms what was observed by TEM for the ferrihydrite phase of S2_air_ sample.

**Figure 6:**
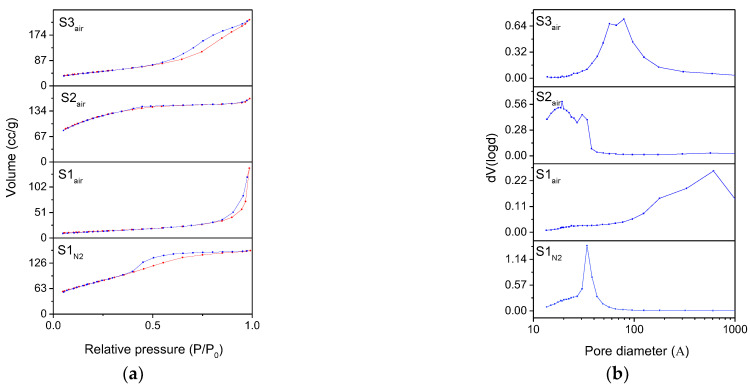
(**a**) N_2_ adsorption (red line)/desorption (blue line) isotherms and (**b**) distributions BJH for desorption branch of the isotherms of the different samples.

The surface area and the porosity of the samples are then evaluated and classified by the nitrogen adsorption measurements, reported in Figure 6, providing other clear evidence of the differences between the samples as well. Concerning the isotherm of the iron oxyhydroxide S1_N2_ sample, it can be related to type IV, typical of mesoporous materials, characterized by a large hysteresis loop associated with capillary condensation as well, which takes place in mesopores. The hysteresis is attributable to H4 type, characteristic of agglomerates of plate-like particles forming slit-like pores, but also internal voids of irregular shape and broad size distribution [36]. The deviation between the adsorption and desorption curves in the initial section can be due to the resolution limit of the instrument (>10 Å) or to limitations related to monolayers [37]. The isotherm of the S1_air_ sample can be ascribed to type IV, but it is characteristic of very little hysteresis due to mesoporous, confirmed by a pore distribution centered in the macropores range. Concerning ferrihydrite in S2 sample and magnetite in S3 sample, they present similar behavior independently from the atmosphere, as also confirmed by the other techniques previously described. Specifically, ferrihydrite S2 samples exhibit a type I(b) isotherm, typical of materials having pores with dimensions in the upper range of the micropore domain, containing both micropores and narrow mesopores (<2.5 nm) [38]. The little adsorption–desorption hysteresis in the range 0.30 < P/P_0_ < 0.60 underlines a very small contribution of mesopores. The isotherm of the magnetite S3 samples can be related to type IV, similarly to that observed in magnetite S1_air_ sample. However, the hysteresis loops underline the differences between the two samples: while in S1_air_ we observe a typical H4 hysteresis loop, S3 exhibits a well-defined hysteresis loop, attributable to the H1 classification, where agglomerates or spherical particles arranged in a fairly uniform way, indicating a relatively high pore size uniformity and facile pore connectivity as well.

The corresponding BJH pore size distributions and calculations are reported in Figure 6b and in Table 3, respectively. The iron oxyhydroxide S1_N2_ sample has a pore size distribution mainly peaked around 4 nm, with pore volume values of 0.25 cc/g. Magnetite S1_air_ sample is instead characterized by a broad pore size distribution, shifted towards the macropores region, with a low presence of mesopores (<50 nm) and revealing the lowest value of pore volume (0.22 cc/g). Confirming their adsorption isotherm, ferrihydrite S2 samples present a bimodal pore size distribution, peaked around 3–4 and 1–2 nm, corresponding to micropores and narrow mesopores and a reduced pore volume of about 0.25 cc/g. Finally, the magnetite S3 samples show a well-defined and bimodal pore distribution around the value of 10 nm, corresponding to the highest value of pore volume (0.36 cc/g) measured between the samples.

Concerning the BET specific surface areas, reported in Table 3, all the samples exhibit considerably high values with respect to the results reported in literature [38,39,40,41,42]. In particular, the BET surface area of the S2_air_ sample is about 420 m^2^/g, while the BET surface area of the S3_air_ sample is about 169 m^2^/g, values extraordinarily higher than the typical values presented in literature for ferrihydrite and magnetite, respectively.

From the characterization analyses, it appears evident that the nature of the reactions combined with our method of synthesis makes the ion exchange process very effective, flexible, and suitable for engineering scopes. In fact, starting from a cheap and similar kind of reagents, we are able to easily switch from one route to another, modulating the characteristics of the iron oxide NPs in order to tailor them with the envisaged application.

### 3.2. Characterization of Iron Oxides Nanoparticles Obtained by Calcination Treatments

**Figure 7:**
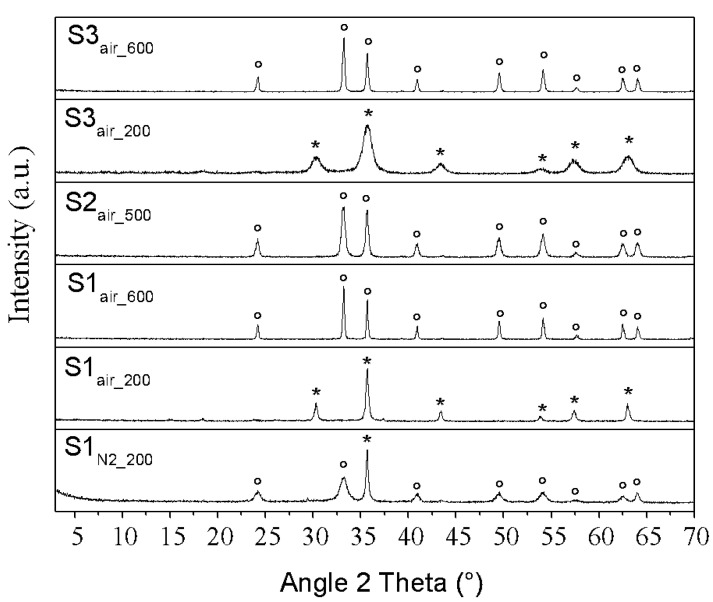
XRD patterns of calcined samples, obtained at different temperatures from different precursors. Legend: * = γ-Fe_2_O_3_ (substoichiometric maghemite), ° = α-Fe_2_O_3_ (hematite).

XRD results of the samples obtained after the calcination treatments of the phases synthesized by the ion exchange process are reported in Figure 7, while the crystallographic parameters of the obtained phases as well as their average D values, evaluated by the Rietveld refinements, are reported in Table S2. In addition, in Figure S3 the comparison between the XRD results, in terms of peaks list, and the ICDD reference patterns is reported as well.

In sample S1_N2_200_, we observed that the iron oxyhydroxide S1_N2_ sample presents the conversion into hematite (α-Fe_2_O_3_, hexagonal crystal structure, ICDD# 00-001-1053) at extraordinarily low temperature conditions (that is, 2 h at 200 °C), characterized by a <D> value of about 3 nm. However, probably due the unavoidable presence of oxygen during the calcination treatment, a maghemite phase is observed too (γ-Fe_2_O_3_, cubic crystal structure, ICDD# 00-004-0755) in a relative amount of 13.9%, as evaluated by Rietveld analysis. In particular, from the Rietveld analysis the obtained cubic maghemite results are substoichiometric in relation to the lower value of its cell parameter with respect to the reference one (ICDD # 00-004-0755).

Differently, the magnetite S1_air_ sample shows, at 200 °C, a complete transformation into pure maghemite, γ-Fe_2_O_3_ (sample S1_air_200_), which in turn transforms in pure hematite increasing temperature up to 600 °C (sample S1_air_600_, ICDD # 00-024-0072). Regarding the ferrihydrite S2 samples, independently from the ambient conditions, we observed a phase transformation only at a temperature of 500 °C, with the formation of pure hematite, with an average <D> value of 20 nm, as shown in the representative XRD pattern of S2_air_500_ sample (ICDD # 00-024-0072). For magnetite S3 samples, we obtained the same results independently from the atmosphere conditions, with the transformation of the initial magnetite into maghemite at a temperature of 200 °C, (sample S3_air_200_), and to hematite at 600 °C, (sample S3_air_600_). In terms of phase transformations, these results are like observed in the S1_air_ sample, since both S3 and S1_air_ precursors are composed of pure magnetite. Nevertheless, the broadening analyses of small crystal sizes for the maghemite and the hematite coming from S3 samples reveals different microstructures, as shown in Figure 7 and in Table S2. Specifically, the maghemite and hematite phases are characterized by an average crystallite size <D> of about 22 and 64 nm if obtained by S1_air_ precursor sample, respect to values of 6 and 42 nm, when the S3 precursor samples are used.

Finally, the analyses of the crystallographic parameters, reported in Table S2, show slight larger cell volumes in the phases characterized by lower D values, denoting greater amounts of lattice defects. In any case, for all the obtained maghemite and hematite phases, the D_hkl_ values are similar for all peaks, denoting a uniform growth of crystal size in all directions [29].

All these results underline a strong influence of the initial precursors on the phase transformations and favoring the transition into hematite, not only due to temperature, but also to different oxidation or dehydration phenomena occurring during the calcination treatments.

**Figure 8:**
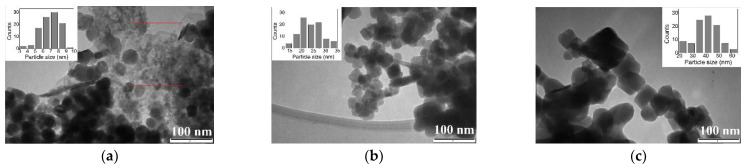

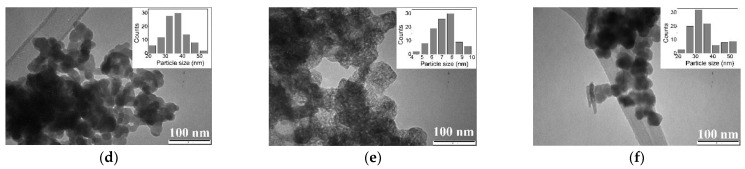
TEM images of calcined samples, obtained at different temperatures: (**a**) hematite/maghemite S1_N2_200_ sample; (**b**) maghemite S1_air_200_ sample; (**c**) hematite S1_air_600_ sample; (**d**) hematite S2_air_500_ sample; (**e**) maghemite S3_air_200_ sample; (**f**) hematite S3_air_600_ sample.

TEM images, reported in Figure 8, show dimensions and morphologies of the calcined particles. We can observe that all the calcined phases have particles in the nano-range, nevertheless characterized by different features one each other. Specifically, S1_N2_200_ sample appears constituted by very small hexagonal nanoparticles, which could be attributed to hematite, having dimensions of less than 5 nm (as marked by in the rectangular insert in Figure 8a), in agreement with the low crystalline hematite phase observed by XRD analysis. Traces of the precursor as well as pseudo-circular particles, with dimensions from 20 to 50 nm, are clearly observed too, attributable to the formation of hexagonal maghemite phase. These particles are indeed similar to those observed in S1_air_200_ sample (Figure 8b), characterized by a pure maghemite phase (as shown in XRD analyses). Concerning the S1_air_600_ sample, TEM images show the presence of nanoparticles characterized by a rhombohedral morphology (Figure 8c), the other typical feature of hematite nanoparticles [43]. The particles in S2_air_500_ sample, shown in Figure 8d, also attributable to hematite by XRD, show a tiny and quasi-spherical shape, related to the hexagonal morphology, frequently observed after the dehydration of ferrihydrite particles and the subsequent gradual structural ordering to hematite structure [44,45,46]. Concerning the S3_air_200_ sample (Figure 8e), it appears constituted by thin lamellae composed of very small nanoparticles (≈7 nm) through a mesoporous assembly characterized by pores less than 10 nm in size. These nanoparticles, associated to maghemite small crystals, transform, at 600 °C, (S3_air_600_ sample), into hematite nanoparticles (Figure 8f), showing a rhombohedral morphology, similarly to what observed for hematite S1_air_600_ nanoparticles, but with smaller dimensions. All these results strongly underline the influence that the initial precursor provides on the formation of the subsequent iron oxides, in relation to dimensions as well as to morphology that is extremely important for the applications.

**Figure 9:**
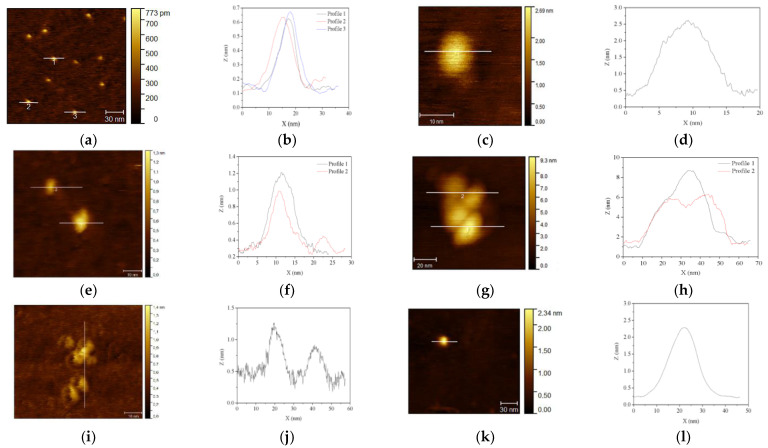
AFM images of calcined samples, obtained at different temperatures: (**a**,**b**) hematite S1_N2_200_ sample, (**c**,**d**) maghemite S1_air_200_ sample, (**e**,**f**) hematite S1_air_600_ sample, (**g**,**h**) hematite S2_air_500_ sample, (**i**,**j**) maghemite S3_air_200_ sample, (**k**,**l**) hematite S3_air_600_ sample.

AFM investigations allow confirming what is observed by TEM, providing further important information as well. Specifically, in S1_N2_200_ sample, we observed very fine and monodisperse particles of about 10 nm length and 0.6 nm height (Figure 9a,b), which can be attributable to the small hexagonal hematite nanoparticles, clearly characterized by monodisperse and spherical shaped particles. A plate-like shape for hematite nanoparticles is also confirmed for the S2_air_500_ sample, but with larger dimensions of about 20 nm length and 4 nm height, (Figure 9g,h). Concerning the particles observed in maghemite S1_air_200_ (Figure 9c,d) and hematite S1_air_600_ samples (Figure 9e,f), they present a circular and an elongated shape, respectively, confirming TEM observations, and both revealing very reduced thickness (about 1–2 nm). In the S3_air_200_ sample, AFM allows confirming and evaluating the dimensions of the small particles that composed the maghemite phase, resulting in about 5 nm length and 0.5 nm height (Figure 9i,j). Finally, tiny single particles of about 20 nm are observed for hematite S3_air_600_ sample (Figure 9k,l).

**Figure 10:**
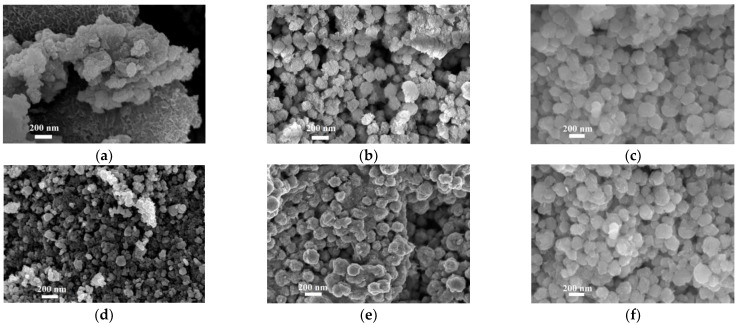
SEM images of the nanoparticles aggregation from different samples: (**a**) hematite/maghemite S1_N2_200_ sample, (**b**) maghemite S1_air_200_ sample, (**c**) hematite S1_air_600_ sample, (**d**) hematite S2_air_500_ sample, (**e**) maghemite S3_air_200_ sample, (**f**) hematite S3_air_600_ sample.

SEM images, reported in Figure 10, show that the nanoparticles obtained after the calcination process form spherical aggregates independently from the precursors and the formed phase. However, hematite S2_air_500_ sample shows smaller aggregates with respect to the other samples, probably due to its smaller dimensions and to the plate-like morphology as well.

**Figure 11:**
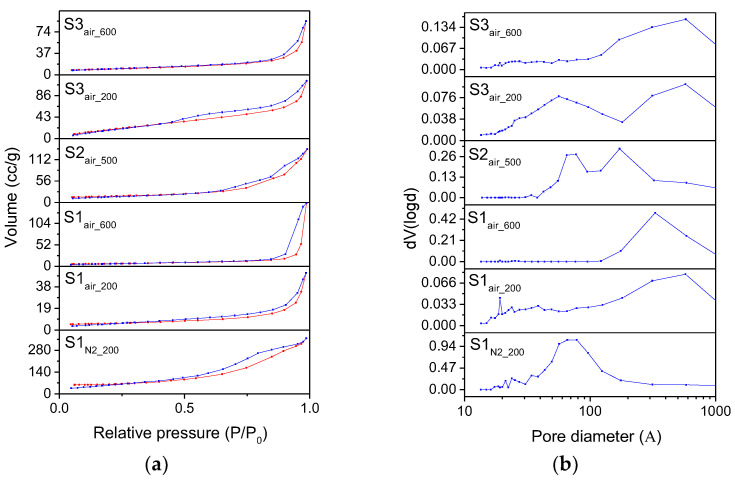
(**a**) N_2_ adsorption/desorption isotherms and (**b**) distributions BJH for desorption branches of the isotherms of different calcined samples.

Finally, in Figure 11a, the nitrogen adsorption isotherms of the calcined samples are shown. The isotherms are all attributable to type IV isotherm, typical of mesoporous materials, as also evidenced by the hysteresis loop between the adsorption and desorption branches. Nevertheless, if the type of isotherms is the same, the hysteresis loops allow distinguishing the samples from one another. Specifically, hematite/maghemite S1_N2_200_ sample exhibits a type H2 hysteresis loop, occurring in highly heterogeneous pore networks [47], and a large monomodal pore distribution around 8 nm. In the S1_air_200_ sample, the maghemite nanoparticles are characterized by a type H1 hysteresis loop, corresponding to the presence of both cylindrical and plate-like pores, with the cylindrical pores having a pore size distribution between 50 and 200 nm (macropores range) and the plate-like pores generally varying in the range 5–50 nm [36,48]. This result is confirmed by the pore size distribution in Figure 11b, confirming the prevalence of macropores, but having a small peak at around 3 nm as well. Similar results are observed for hematite S1_air_600_ and S3_air_600_ samples, but limiting the hysteresis to the presence of cylindrical and relatively larger pores, as verified in the corresponding pore size distribution. A H1 type hysteresis loop can be also remarked for hematite S2_air_500_ sample, in this case associated with a bimodal pore distribution in the mesopores region, around 8 nm and around 20 nm, which defines only the presence of plate-like mesopores. Differently from the other samples, the hysteresis loop of maghemite obtained in the S3_air_200_ sample is a H3 type, with the characteristic step down at relative pressures ≈ 0.4. This kind of hysteresis is usually related to non-rigid aggregates of plate-like particles or assembly of slit-shaped pores determining two classes of pores size, in the range 2–50 and 50–100 nm, due to slit-/plate-like particles, respectively [47,49]. The bimodal pore size distribution is confirmed by the pore distribution reported in Figure 11b, where this maghemite sample exhibits two peaks around 8 and 80 nm.

The results of the BET and BJH calculations of all the calcined samples, in terms of surface area and pore diameters, are finally reported in Table 4.

As expected from the XRD and TEM analyses, the S2_N2_200_ and the S2_air_500_ samples exhibit the highest values of specific surface area. Moreover, it is to be remarked these BET surface areas are higher than most of the experimental results reported, up to today, in literature for hematite [50].

## 4. Conclusions

Iron oxides NPs include several kinds of compounds covering a lot of applicative fields. For their peculiar magnetic properties, nontoxicity and biodegradability, the interest is continuously increasing in biomedicine, sensors, as well as in environmental remediation.

The transition from lab synthesis to large scale applications is of paramount importance for their tangible uses in all these applications. Nevertheless, up to now, scalable as well as eco-friendly NPs productions represent a global challenge due to the complexity of set-up, high temperatures/pressure involved, needs of multistep processes to purify the product from organic or secondary substances, longtime synthesis, and production of waste, also determining high selling costs.

In order to answer a need for production on a large scale at low environmental impact, we defined a new method to obtain a production of iron oxide NPs with low costs, using non-toxic and cheap precursors, starting from a rapid, green, and sustainable approach.

In this paper, we present a sustainable, one-step method based on an ion exchange process, starting with non-toxic, cheap, or renewable precursors, and characterized by rapid times, allowing the production of the main applicable types of pure iron oxide/oxyhydroxide NPs (related to ferrihydrite, δ-FeOOH, magnetite, hematite, and maghemite), with a high purity, small particles dimensions (<30 nm), and high specific surface area, with yields of production > 90%. In addition, the proposed process represents a very powerful, flexible, and suitable method for a sustainable production of iron-based NPs. In fact, starting from the same reagents, we are able to switch easily from the synthesis of one compound to the other, modulating their characteristics in order to engineer the NPs and tailor them with the required applications.

## Figures and Tables

**Table 1:** Expected iron compounds that can be obtained by the innovative one-step synthesis method. The reaction atmosphere (N_2_, air), the chemical reactions, and the oxidation state of the initial iron reagents (Fe(II); Fe(III); Fe(II) + Fe(III)) are correspondingly reported.

Suspension Sample	Oxidation State of Initial Reagents	Atmosphere	Expected Compounds	Reaction
S1_N2_	Fe (II)	N_2_	δ-FeOOH	5
S1_air_		air	Fe_3_O_4_	4
S2_N2_	Fe(III)	N_2_	Fe_5_HO_8_⋅4H_2_O	6
S2_air_		air		6
S3_N2_	Fe(II) + Fe(III)	N_2_	Fe_3_O_4_	7
S3_air_		air		7

**Table 2:** pH values, reduction of the chloride concentration (ΔCC), and residual chloride concentration (RCC) at different times in relation to each synthesis. The amounts of NPs produced in 10 min and the final yield of the production (Y) directly obtained by the ion exchange method are reported too, in correspondence to each expected chemical reaction (see Table 1).

Suspension Sample	pH 0 min	pH 10 min	ΔCC (%) 1 min	ΔCC (%) 10 min	RCC (mg/L)	NPs Produced in 10 min (g)	Yield (%)
S1_N2_	2.5	9.1	96.8	99.1	29.7	44	92
S1_air_	2.5	7.5	96.9	99.3	27.3	45	94
S2_N2_	1.6	7.5	99.2	99.9	21.4	41	94
S2_air_	1.6	7.5	99.1	99.9	21.6	42	95
S3_N2_	1.6	7.5	98.2	99.5	24.5	36	94
S3_air_	1.6	7.5	98.3	99.7	23.4	37	96

**Table 3:** BET specific surface areas of the samples synthesized by the ion exchange process. Pore volume and pore diameters from BJH calculations are reported too.

	Iron Oxyhydroxide S1_N2_	Magnetite S1_air_	Ferrihydrite S2_air_	Magnetite S3_air_
BET surface area (m^2^/g)	271	46	420	169
BJH pore diameter Dv(d) (nm)	3.41	2.38	1.50	5.69
BJH pore volume (cc/g)	0.25	0.22	0.24	0.36

**Table 4:** Specific surface areas calculated by BET method for N_2_ adsorption isotherms of the samples: S1_N2_200_, S1_air_200_, S1_air_600_, S2_air_500_, S3_air_200_, and S3_air_600_. Pore volume and pore diameters from BJH calculations are reported too.

Starting Sample	S1_N2_	S1_air_		S2_air_	S3_air_	
Calcination temperature	200 °C	200 °C	600 °C	500 °C	200 °C	600 °C
Obtained Compound	Hematite	Maghemite	Hematite	Hematite	Maghemite	Hematite
BET surface area (m^2^/g)	205	20	22	58	37	34
BJH pore diameter Dv(d) (nm)	5.67	1.91	32.99	6.53	3.43	3.91
BJH pore volume (cc/g)	0.60	0.08	0.24	0.22	0.21	0.14

## Supplementary Materials

The following are available online at https://www.mdpi.com/2079-4991/11/3/798/s1: Table S1: Crystallographic parameters and average crystallite size <D> related to the crystalline iron phases of the one-step syntheses, as evaluated by Rietveld refinements. Only the results related to S2_N2_ and S2_air_ samples are not reported due to their amorphous behavior; Table S2: Crystallographic parameters and average crystallite size <D> related to the calcined iron phases, resulting by Rietveld refinement; Figure S1: Comparison between the XRD results and the ICDD reference patterns. (a) S1_N2_ sample, (b) S1_air_ sample, (c) S3_air_ sample; Figure S2: Photographic illustration on the synthetic route for the production of δ-FeOOH in powder form; Figure S3: Comparison between the XRD results and the ICDD reference patterns. (a) S1_N2_200_ sample, (b) S1_air_200_ sample, (c) S1_air_600_ sample, (d) S2_air_500_ sample, (e) S3_air_200_ sample, (f) S3_air_600_ sample.
