# Supplementary material for: New Sustainable, Scalable and One-Step Synthesis of Iron Oxide Nanoparticles by Ion Exchange Process
Source: Nanomaterials (Basel). 2021 Mar 20;11(3):798. doi: 10.3390/nano11030798 (PMC8004010; doi:10.3390/nano11030798)
Supplement: Supplementary file 1 [file nanomaterials-11-00798-s001.pdf]

Supplementary Materials

New Sustainable, Scalable and One-Step Synthesis of Iron Oxide Nanoparticles by Ion Exchange Process

Ludovico Macera [1], Valeria Daniele [1,*], Claudia Mondelli [2], Marie Capron [3,4] and Giuliana Taglieri [1]

Table S1. Crystallographic parameters and average crystallite size <D> related to the crystalline iron phases of the one-step syntheses, as evaluated by Rietveld refinements. Only the results related to $S2_{N2}$ and $S2_{air}$ samples are not reported due to their amorphous behaviour.

Sample	Phase	Crystal System	a (Å)	b (Å)	c (Å)	Cell Volume (Å^3)	<D> (nm)
$S1_{N2}$	δ-FeOOH	hexagonal	2.95747	2.95747	4.55283	34.48675	5
$S1_{air}$	Fe_3O_4	cubic	8.38025	8.38025	8.38025	588.5333	29
$S3_{air}$	Fe_3O_4	cubic	8.38158	8.38158	8.38158	588.8129	8

Table S2. Crystallographic parameters and average crystallite size <D> related to the calcined iron phases, resulting by Rietveld refinement.

Sample	Phase	Crystal System	a (Å)	b (Å)	c (Å)	Cell Volume (Å^3)	<D> (nm)
$S1_{N2\_200}$	α-Fe_2O_3	hexagonal	5.04865	5.04865	13.88428	306.4819	3
	γ-Fe_2O_3	cubic	8.346	8.346	8.346	581.3465	16
$S1_{air\_200}$	γ-Fe_2O_3	cubic	8.33975	8.33975	8.33975	580.0414	22
$S1_{air\_600}$	α-Fe_2O_3	hexagonal	5.03431	5.03431	13.74726	301.7362	64
$S2_{air\_500}$	α-Fe_2O_3	hexagonal	5.03424	5.03424	13.7532	301.8578	20
$S3_{air\_200}$	γ-Fe_2O_3	cubic	8.34463	8.34463	8.34463	581.0602	6
$S3_{air\_600}$	α-Fe_2O_3	hexagonal	5.03579	5.03579	13.75243	302.0264	42

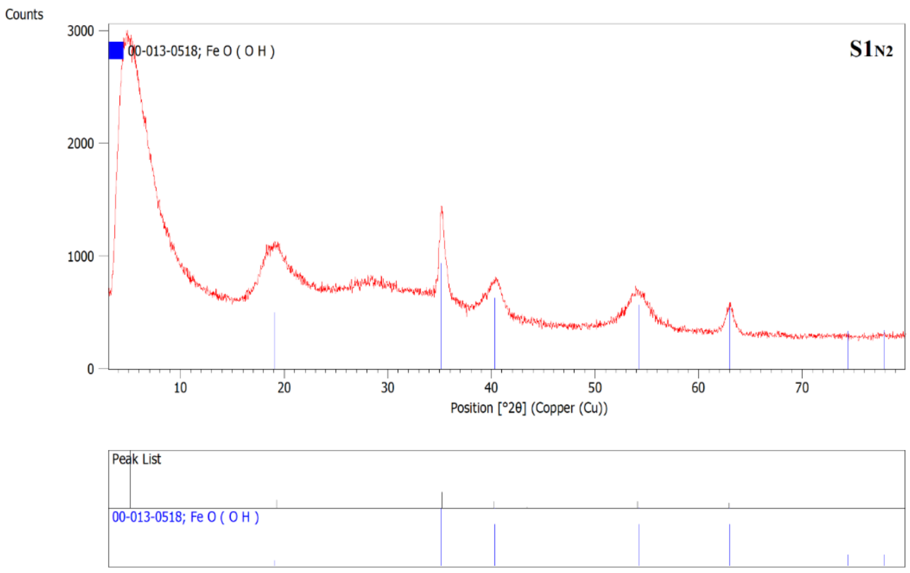

(a)

(b)

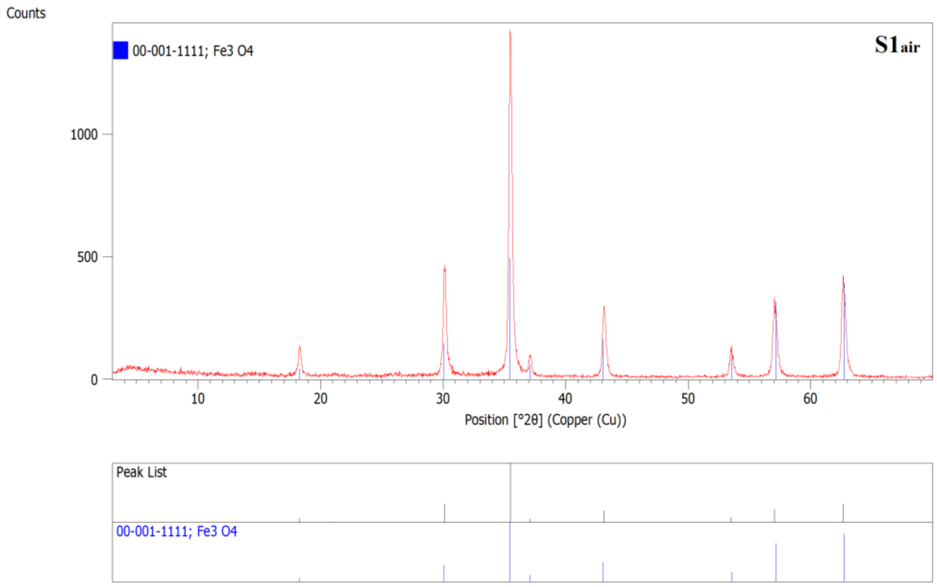

(c)

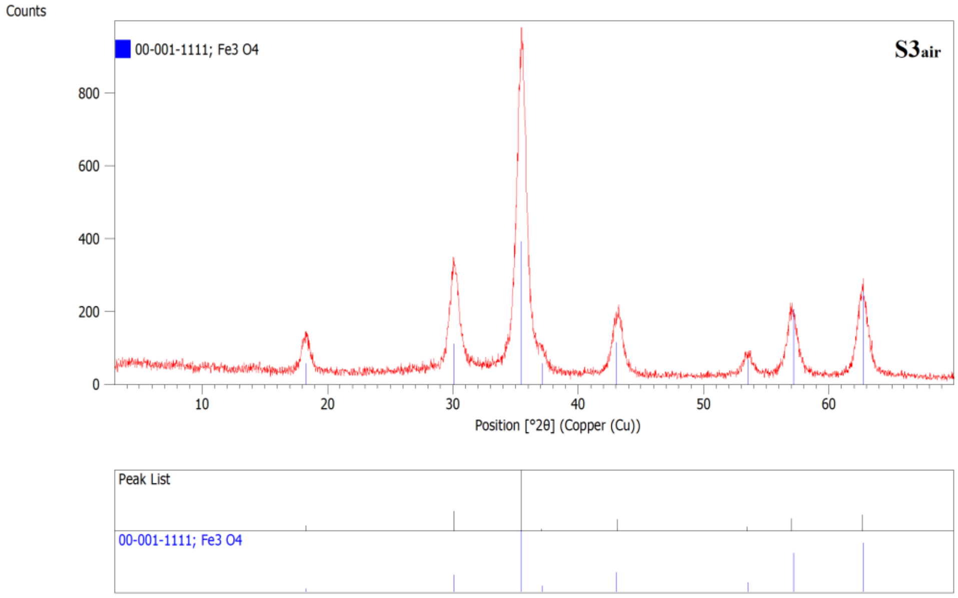

Figure S1. Comparison between the XRD results and the ICDD reference patterns. (**a**) $S1_{N2}$ sample, (**b**) $S1_{air}$ sample, (**c**) $S3_{air}$ sample.

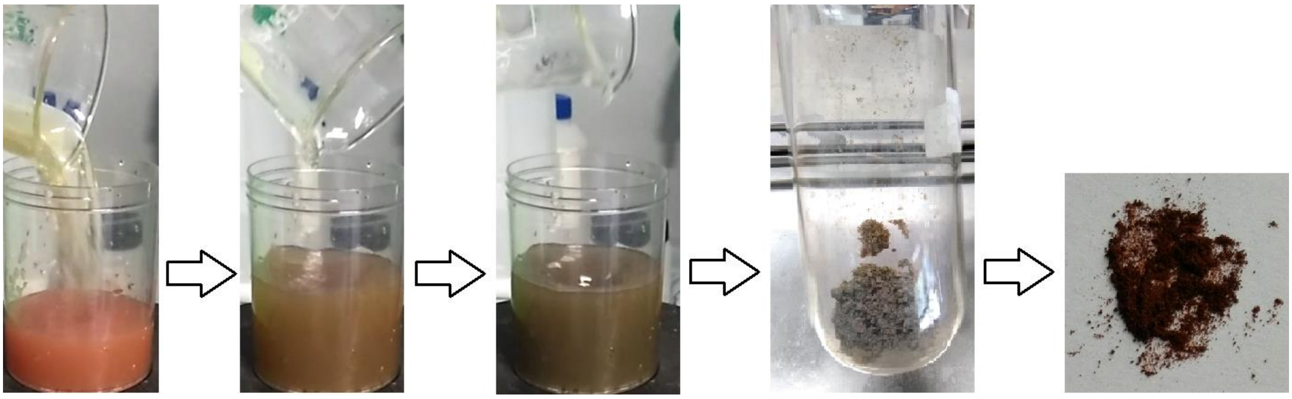

Figure S2. Photographic illustration on the synthetic route for the production of δ-FeOOH in powder form.

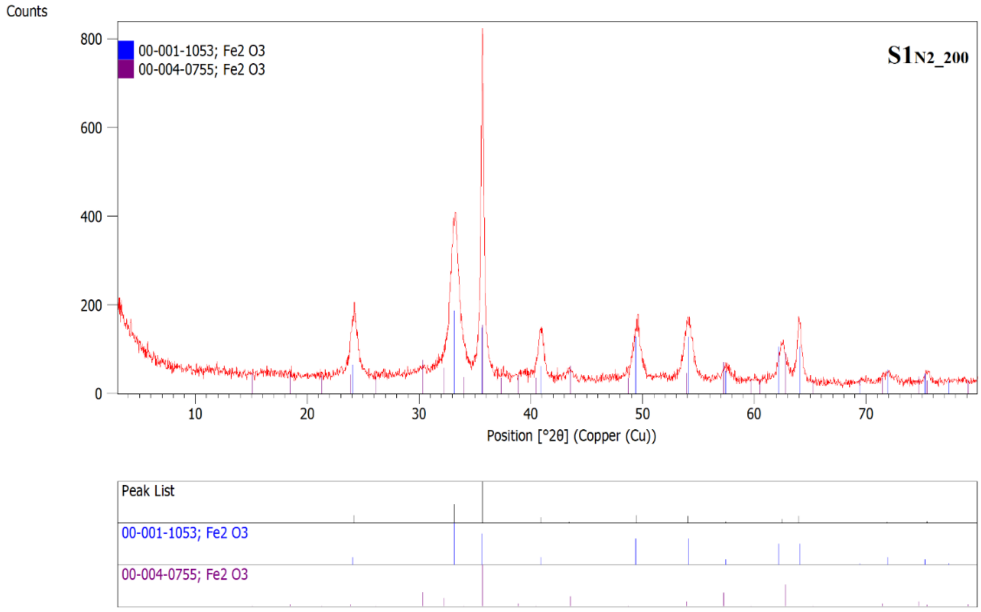
Counts
800
600
400
200
0
00-001-1053; Fe2 O3
00-004-0755; Fe2 O3
S1N2_200
10
20
30
40
50
60
70
Position [°2θ] (Copper (Cu))
Peak List
00-001-1053; Fe2 O3
00-004-0755; Fe2 O3

(a)

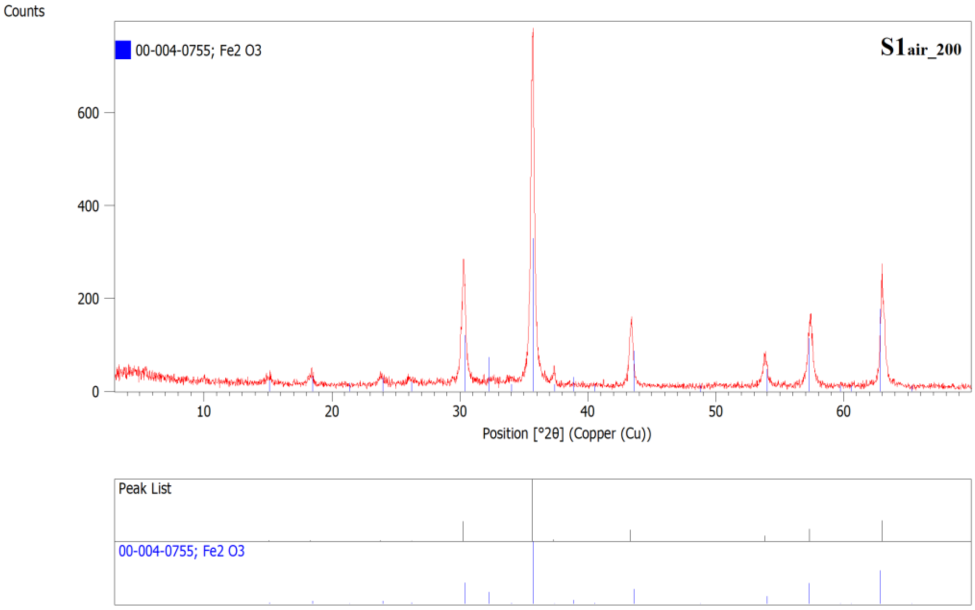
Counts
600
400
200
0
00-004-0755; Fe2 O3
S1air_200
10
20
30
40
50
60
Position [°2θ] (Copper (Cu))
Peak List
00-004-0755; Fe2 O3

(b)

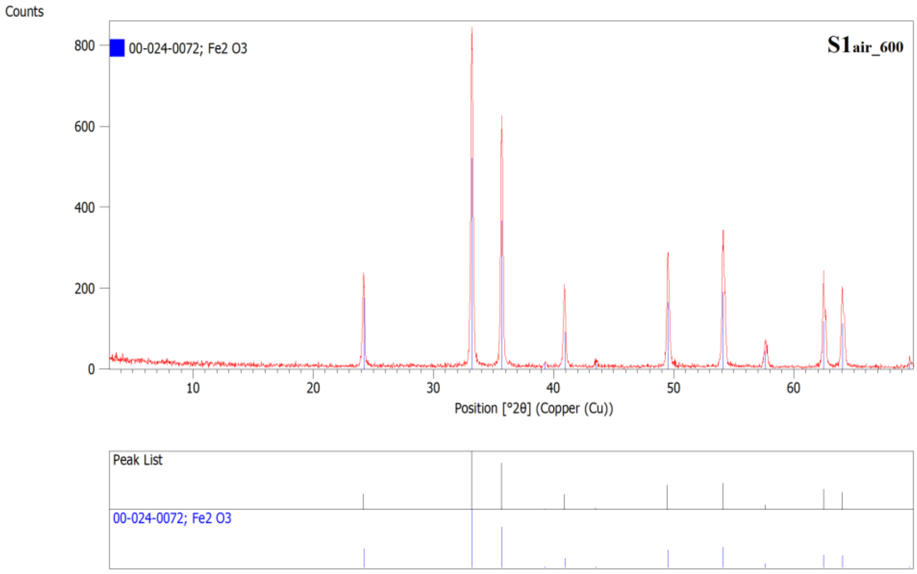
Counts
800
600
400
200
0
00-024-0072; Fe2 O3
S1air_600
10
20
30
40
50
60
Position [°2θ] (Copper (Cu))
Peak List
00-024-0072; Fe2 O3

(c)

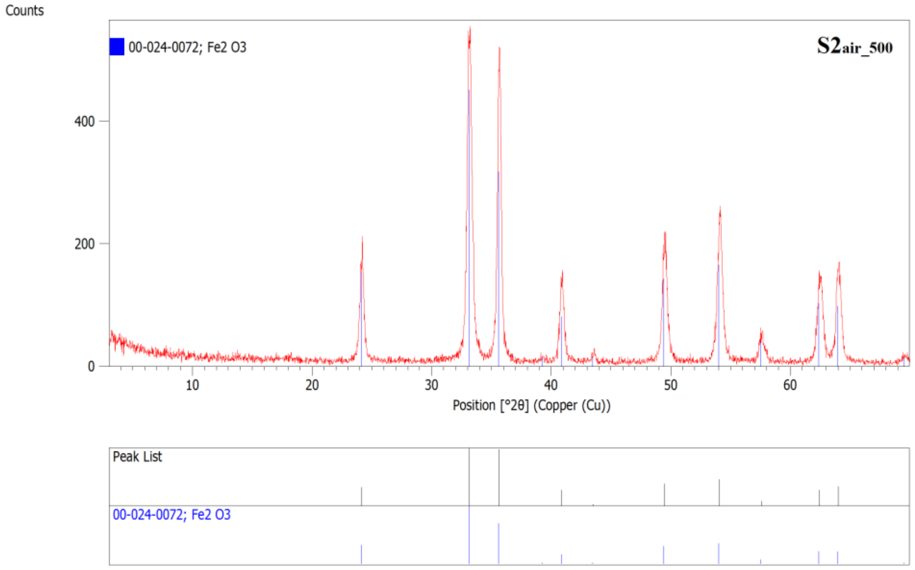

(d)

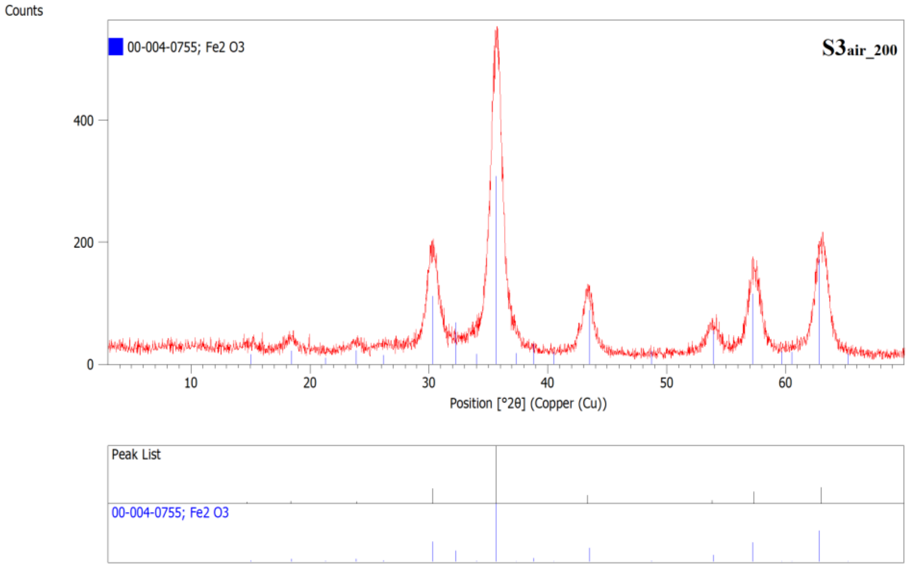

(e)

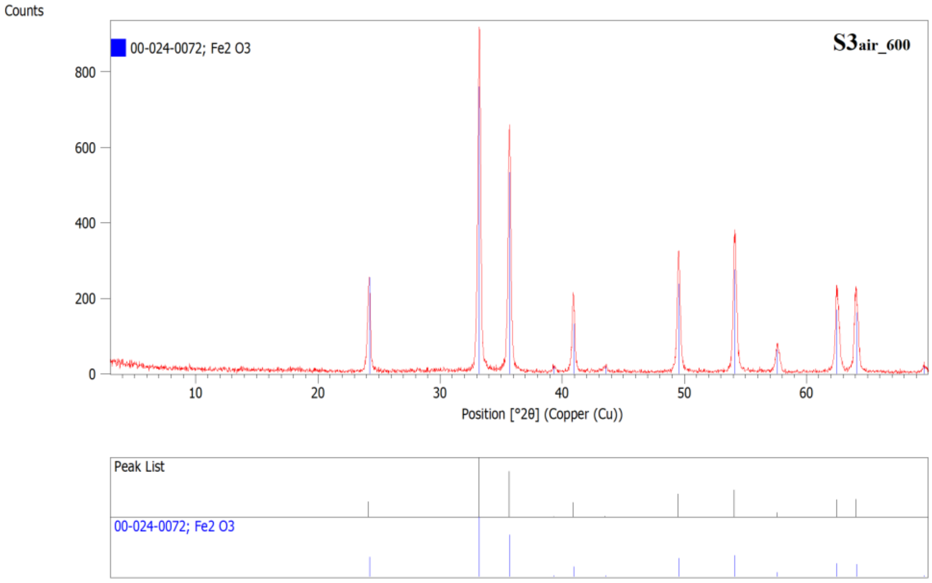

(f)

Figure S3. Comparison between the XRD results and the ICDD reference patterns. (**a**) $S1_{N2\_200}$ sample, (**b**) $S1_{air\_200}$ sample, (**c**) $S1_{air\_600}$ sample, (**d**) $S2_{air\_500}$ sample, (**e**) $S3_{air\_200}$ sample, (**f**) $S3_{air\_600}$ sample.
